# Pediatric torticollis: clinical report and predictors of urgency of 1409 cases

**DOI:** 10.1186/s13052-024-01653-6

**Published:** 2024-04-24

**Authors:** Umberto Raucci, Marco Roversi, Alessandro Ferretti, Valerio Faccia, Giacomo Garone, Fabio Panetta, Carlo Mariani, Eloisa Rizzotto, Antonio Torelli, Giovanna Stefania Colafati, Angelo Gabriele Aulisa, Pasquale Parisi, Alberto Villani

**Affiliations:** 1 Institute of Child and Adolescent Health, Bambino Gesù Children’s Hospital, IRCCS, Rome, Italy; 2 Clinical Trial Area, Development and Implementation of Drugs, Vaccines, and Medical Devices for Pediatric Use, Bambino Gesù Children’s Hospital, IRCCS, Rome, Italy; 3 PhD Program in Immunology, Molecular Medicine and Applied Biotechnology, University of Rome Tor Vergata, Rome, Italy; 4 Chair of Pediatrics, Department of Neuroscience, Mental Health and Sensory Organs (NESMOS), Faculty of Medicine and Psychology, Sant’ Andrea Hospital, Sapienza University of Rome, Rome, Italy; 5 Neurology, Epilepsy and Movement Disorders Unit, Full Member of European Reference Network on Rare and Complex Epilepsies, EpiCARE, Bambino Gesù Children’s Hospital, IRCCS, Rome, Italy; 6 General Pediatrics and Emergency Department 2nd Level, Bambino Gesù Children’s Hospital, IRCCS, Rome, Italy; 7 Residency School of Pediatrics, University of Rome Tor Vergata, Rome, Italy; 8 Department of Imaging, Bambino Gesù Children’s Hospital, IRCCS, Rome, Italy; 9 Orthopaedics and Traumatology Division, Bambino Gesù Children’s Hospital, IRCCS, Rome, Italy; 10 Department of Human Sciences, Society and Health, University of Cassino and Southern Lazio, 03043 Cassino, Italy; 11 Systems Medicine Department, University of Rome Tor Vergata, Rome, Italy; 12 General Pediatrics and ED 2nd Level, Bambino Gesù Children’s Hospital IRCCS, Rome, Italy

**Keywords:** Torticollis, Emergency, Children, Pediatrics, Neck complaints

## Abstract

**Background:**

To date, the etiology and risk factors of torticollis are still poorly defined in the pediatric literature. Especially in the Emergency Department (ED) scenario, it is critical to reliably distinguish benign and transient conditions from (potentially) life-threatening disorders. This study describes the clinical characteristics of a large sample of children with torticollis. The aim of our study was to detect epidemiology, etiology and predictive variables associated with a higher risk of life-threatening conditions in acute torticollis.

**Methods:**

We conducted a pediatric retrospective study of acute torticollis over a 13-year period referred to the ED of a tertiary pediatric Hospital. We reported the characteristics in the overall sample and in two subgroups divided according to urgency of the underlying condition. Furthermore, we developed a multivariate model aimed at identifying the main clinical predictors of the need for urgent care.

**Results:**

1409 patients were analyzed (median age 5.7 years, IQR 5.8). A history of trauma was present in 393 patients (27.9%). The symptom most frequently associated with torticollis were pain (83.5%). At least one pathological finding was found in 5.4 to 7.9% of patients undergoing further imaging. Hospitalization was required in 11.1% of cases (median duration 4 days). The most frequent etiologies of torticollis were postural cause (43.1%), traumatic (29.5%), and infective/inflammatory (19.1%). A longer time from onset of torticollis and the presence of headache or vomiting were strongly correlated with an underlying urgent condition, after adjusting for the other clinically and statistically significant variables in the bivariate analysis.

**Conclusion:**

Our study shows that an urgent condition most commonly occur in patients presenting with history of trauma or headache, vomiting and torticollis for more than 24 h should undergo further diagnostic evaluation and short-term follow-up, restricting invasive or expensive investigations to patients with clinical suspicion of an underlying harmful condition.

**Supplementary Information:**

The online version contains supplementary material available at 10.1186/s13052-024-01653-6.

## Background

Torticollis is an alteration of neck motility, characterized by the pathologic contraction or shortening of the sternocleidomastoid muscle (SCM), with lateral rotation of the cervical spine, accompanied by a contralateral head tilt, associated or not with pain. Major causes of this condition include abnormal movements, trauma, infection/inflammation, congenital abnormalities, ophthalmologic, gastrointestinal, neuromuscular diseases, and tumors [1]. Incidence and clinical presentation of torticollis in children have not yet been consistently described, primarily due to the heterogenous etiological background. Torticollis is traditionally classified according to its congenital or acquired nature. Congenital muscular torticollis is the result of asymmetric shortening of the SCM muscle caused by altered intrauterine growth of the muscle or direct trauma at birth that results in a palpable hematoma which causes fibrotic shortening of the muscle. An eye defect (congenital or acquired) may also cause a compensatory rotation of the head or an extension or flexion movement of the head to facilitate binocular vision [2]. On the other side, the underlying causes of an acquired torticollis are varied, including trauma and infection, but also fewer uncommon causes, including neurological disorders such as dystonia, central nervous system tumors, and Grisel syndrome [3].

In the current literature, two major studies have provided insights into the causes of pediatric torticollis. The study by Pharisa et al. identified three main causes of torticollis in 170 children under the age of 16 years who were admitted: traumatic (62%), infective (19.6%), and postural (17.6%) [4]. The more recent retrospective study by Starc et al. investigated 392 pediatric patients, revealing a different distribution of causes: postural (61%), infective (19.4%), and traumatic (16.3%) [5]. In the same study, an attempt was made to identify red flags for hospitalization outcome. These red flags included age below 5 years, second admission to the emergency department (ED) for the same issue, presence of fever, pharyngodynia or headache. In a recent systematic review of 45 articles, 37 out of the 95 included patients (39%) had torticollis as the first and only presenting symptom of a brain tumor [6]. Numerous case reports in the literature describe other unusual causes of pediatric torticollis [3, 7–16]. Because of these conflicting and sparse data, a very recent review [17] and best practice guideline [18] suggest that there is still ample room for research.

This study aims to describe the demographic and clinical characteristics of pediatric patients referred to our center for torticollis, to assess the different etiologies of torticollis, and to identify risk factors that may provide guidance to the emergency pediatrician.

## Methods

We conducted a retrospective review of the medical records of all children aged less than 18 years admitted for torticollis to the ED of the Bambino Gesù Children’s Hospital, IRCCS (Rome, Italy), a tertiary pediatric hospital, between January 2009 and December 2021. All ED clinical charts reporting the words “torticollis” and/or “cervical” in any given section were screened for relevance. Patients in whom the diagnosis of torticollis was not confirmed in the following work-up or was already attributed to a known medical condition were excluded. Similarly, patients diagnosed with different syndromes of neck pain and abnormal posturing (e.g., neck stiffness due to meningeal irritation) were excluded. The following data were extracted from the clinical charts: age; sex; triage code; time from onset to referral; clinical presentation according to medical history and physical examination; laboratory workup, medical imaging; specialist consultation; treatment of torticollis; hospitalization; sequelae. The etiologies of torticollis were grouped according to the final diagnoses. We then divided the causes of torticollis according to the risk of threat to the individual’s life or health into urgent and non-urgent conditions and compared the aforementioned variables across these two groups. In more detail, the causes of urgent torticollis were: traumatic with fracture or atlanto-epistrophic dislocation; retropharyngeal abscess and Grisel’s syndrome among the infective/inflammatory causes; all neurologic causes excluding benign paroxysmal torticollis and idiopathic/genetic (non-structural) cervical dystonia; all oncologic causes; other causes that pose a risk to the individual *quoad vitam* or *quoad valetudinem*. Relevant and statistically significant variables from univariate and bivariate analysis were entered into a multivariate model aimed at identifying the main clinical predictors of urgent torticollis.

Statistical analysis was performed using the Statistical Package for Social Sciences software (SPSS, version 23.0). Continuous variables were reported in medians, interquartile range, 5th and 95th percentiles and were compared with the Mann-Whitney U test. Categorical variables were reported in absolute numbers and relative percentages and were compared with the Chi-square test. Logistic regression analysis was performed by adopting the clinical urgency of the baseline condition as the dependent variable and the clinically and statistically significant variables at bivariate analysis as the independent variable. The result of statistical tests was considered significant for a p-value less than or equal to 0.05. The study was approved by the Ethics Committee of the Bambino Gesù Children’s Hospital, IRCCS, Rome, Italy.

## Results

**Fig. 1:**
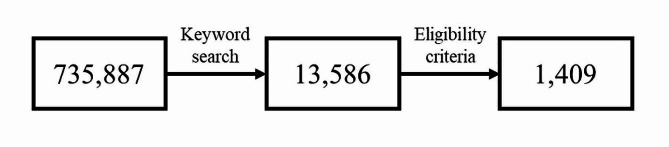
Study flowchart

During this 13-year period, 735,887 admissions to our ED were recorded. Through keyword search, 13,586 medical records were obtained. Of these, only 1409 records met the study eligibility criteria (Fig. 1), with an observed incidence of torticollis presentation of 19 cases per 10,000 accesses.

The clinical and demographic characteristics of the study patients are outlined in Table 1.

**Table 1:** Characteristics of study sample*

Total	1409
Age (years) – median (IQR; 5° − 95°)	5.7 (5.8; 0.5–13.9)
Males – no. (%)	757 (54)
Triage - Code Red – no. (%) - Code Yellow/Orange/Blue – no. (%) - Code Green – no. (%) - Code White – no. (%)	0 (0) 130 (9.2) 1114 (79.1) 163 (11.6)
History of trauma – no. (%)	393 (27.9)
Time from onset to referral (days) – median (IQR; 5° – 95° centile) Time from onset to referral (< 24 h) – no. (%)	0 (0; 0–14) 822 (58.3)
Clinical presentation – no. (%) - Head deviation - to the right - to the left - Pain - Sore throat - Fever (> 38 °C) - Cervical tumefaction/lymphadenopathy - Headache - Earache - Vomiting - Dizziness/vertigo - Other	1179 (83.7) 434 (36.8) 745 (63.2) 1176 (83.5) 225 (16.0) 159 (11.3) 90 (6.4) 72 (5.1) 69 (4.9) 44 (3.1) 19 (1.3) 125 (8.9)
Laboratory workup - WBC (cells/µl) – median (IQR; 5° – 95°) - Neutrophils (%) – median (IQR; 5° – 95°) - Leukocytosis – no. (%)* - CRP ≥ 0.5 mg/dl – no. (%)*	9520 (5550; 5110–19,390) 57.7 (25.5; 23.2–83.5) 68 (30.9) 106 (49.8)
Medical imaging – no. (%) - Radiography with findings - CT scan with findings - Ultrasound with findings - MRI with findings	663 (47.1) 395 (28.0) 82 (20.8) 175 (12.4) 82 (46.9) 165 (11.7) 111 (67.3) 76 (5.4) 42 (55.3)
Specialist consultation – no. (%) - Orthopedic - Neurologist - Otolaryngologist - Neurosurgeon - Ophthalmologist - Oncologist - Anesthesiologist - Other	359 (25.5) 231 (16.4) 112 (7.9) 49 (3.5) 37 (2.6) 35 (2.5) 16 (1.1) 7 (0.5) 29 (2.1)
Treatment of torticollis – no. (%) - Ibuprofen - Acetaminophen - Antibiotics - Cervical collar - Surgery – no. (%)	1255 (89.1) 1121 (79.6) 111 (7.9) 125 (8.9) 806 (57.2) 18 (1.3)
Hospitalization – no. (%) Hospitalization (days) – median (IQR; 5° − 95°) Sequelae – no. (%)	156 (11.1) 4 (4; 1–22) 29 (2.1)

*The percentages were calculated accounting for missing values

The median age of patients was 5.7 (IQR 5.8; 5th-95th, 0.5–13.9) years. Most patients were male (54%), with a female-to-male ratio of 1:1.2. Access priority was considered intermediate/high based on triage (code red-yellow until 2019, red-orange-blue from 2020 onward) in 130 patients (9.2%). A history of trauma was present in 393 patients (27.9%). The median time from onset of torticollis to DEA admission was less 24 h (range 0–14 days), with 822 children brought to the ED in the first 24 h after the onset of torticollis. The side of head deviation was specified in 1179 patients (83.7%), divided into leftward in 745 (63.2%) and rightward deviation in 434 (36.8%) children. The signs and symptoms most frequently reported were pain (83.5%), pharyngodynia (16%), fever (11.3%) and laterocervical tumefaction/lymphadenopathy (6.4%). Less frequently encountered symptoms included headache (5.1%), earache (4.9%), vomiting (3.1%), and dizziness (1.3%). A complete blood count was available for 220 patients. The median leukocyte count of these patients was 9520/µl (with a median neutrophil percentage of 57.7%) but 30.9% of the patients had leukocytosis. The C-reactive protein assay was performed in 213 patients, and 106 (49.8) reported a value above 0.5 mg/dl. Imaging was performed in 663 patients (47.1%). Of these, 28%, 12.4% and 11.7% of patients underwent radiography, CT or ultrasound of the head and neck region, respectively. At least one pathological finding was found in 5.4 to 7.9% of these exams. An MRI was performed in a minority of patients (5.4%). Consultation with a specialist was necessary in 359 cases (25.5%), mainly an orthopedic (16.4%), followed by a neurologist (7.9%). Treatment included prescription of ibuprofen in 1121 patients (79.6%), antibiotics in 125 (8.9%) and acetaminophen in 111 (7.9%). A cervical collar was applied in 806 patients (57.2%). Eighteen patients (1.3%) required surgery. Hospitalization was required in 156 cases (11.1%), with a median duration of 4 days (IQR 4, 5th-95th 1–22). Most patients were discharged without further sequelae (97.9%).

The etiology of torticollis by main categories is shown in Table 2.

**Table 2:** Etiology of torticollis

	No. (%)
Postural	607 (43.1)
Traumatic - Head/cervical trauma - C1-C2 subluxation - Fracture - Abnormal movement - C1-C2 subluxation	415 (29.5) 260 (62.7) 17 (6.5) 5 (1.9) 155 (37.3) 12 (7.7)
**Infective/inflammatory** - Pharyngo-tonsillitis - Cervical lymphadenitis - Otitis media - Post–tonsillectomy/adenoidectomy - Retropharyngeal abscess - Griesel syndrome - Kawasaki disease	**269 (19.1)** 129 (48.0) 71 (26.4) 35 (13.0) 16 (5.9) 9 (3.3) 8 (3.0) 1 (0.4)
**Neurologic** - Benign paroxysmal torticollis - Dystonic drug reaction - Genetic or likely genetic cervical dystonia - Hydrocephalus** - Myelitis - Arachnoid cyst - Arnold–Chiari malformation - Pseudotumor cerebri	**46 (3.3)** 34 (73.9) 3 (6.5) 2 (4.3) 2 (4.3) 2 (4.3) 1 (2.2) 1 (2.2) 1 (2.2)
**Congenital** - Muscular torticollis - Postural deformation/plagiocephaly - Klippel–Feil syndrome	**35 (2.5)** 27 (77.2) 5 (14.3) 3 (8.6)
**Oncologic** - Spinal/brain stem tumor - Langherans cell histiocytosis - Posterior fossa tumor - Bone tumor - Glioma - Giant cell granuloma of the mandible	**17 (1.2)** 5 (29.4) 4 (23.5) 3 (17.7) 2 (11.8) 2 (11.8) 1 (5.9)
**Other** *Ocular* - Strabismus *Osteo-articular* - Acute intervertebral disk calcification - Fibromyalgia - Idiopathic juvenile arthritis - Chronic nonbacterial osteomyelitis *Psychogenic* - Psychic disturbance *Miscellanea* - Vertebral angioma - PTLD*** - Sickle cell disease	**20 (1.4)** 6 (30) 3 (15) 2 (10) 2 (10) 1 (5) 3 (15) 1 (5) 1 (5) 1 (5)

*Only 220 values available for WBC and N, only 212 values available for CRP

**Associated with Dandy Walker malformation and intraventricular hemorrage of MAV

***Post-transplant lymphoproliferative disorder causing a cervical lymphadenopathy

A postural cause was recognized in 607 (43.1%) of patients. For 415 patients (29.5%) torticollis was attributed to a traumatic cause. Of these, 62.7% suffered direct trauma to the head-neck district, resulting in atlantoaxial subluxation in 17 patients (6.5%) and vertebral fracture in 5 patients (1.9%). Abnormal cervical motion (sprain, whiplash, hyperextension, etc.) caused torticollis in 37.3% of patients, resulting in atlanto-axial subluxation in 12 patients (7.7%). No vertebral fractures were documented as a result of abnormal neck movements. An infective/inflammatory etiology involved 19.1% of patients, with pharyngo-tonsillitis as the main cause (48%), followed by laterocervical lymphadenitis (26.4%), and otitis media (13%). The etiology of torticollis was neurological in 46 patients (3.3%), with benign paroxysmal torticollis being the most frequent cause (73.9%). Secondary causes were dystonic drug reactions (6.5%) and dystonia with a probable genetic basis (4.3%). There were 35 patients with congenital torticollis (2.5%), caused by muscular contracture (77.2%), plagiocephaly/postural deformity (14.3%) and Klippel-Feil syndrome (8.6%). Different tumor types caused oncological torticollis in 17 patients (1.2%), mainly bone marrow or brainstem tumor in 5 patients (29.4%), Langerhans cell histiocytosis in 4 (23.5%), posterior cranial fossa tumor in 3 (17.7%). The remaining causes of torticollis in 20 patients (1.4%) included osteoarticular/rheumatologic disorders in 8 patients (40%) and strabismus in 6 (30%).

The comparison between patients with and without an urgent condition underlying the torticollis is shown in Table 3.

**Table 3:** Comparison of patients with an urgent and non–urgent condition*

Total	Urgent	Non-urgent	p–value
	*n* = 78	*n* = 1331	
Age (years) – median (IQR; 5° − 95°)	6.9 (5.0; 1.5–13.1)	5.7 (5.8; 0.5–14.0)	**0.027**
Males – no. (%)	47 (60.3)	621 (46.7)	0.234
Higher priority (code Blue or higher) – no. (%)	29 (37.7)	101 (7.6)	**< 0.001**
History of trauma – no. (%)	32 (41.0)	362 (27.2)	**0.008**
Time from onset to referral (days) – median (IQR; 5° – 95° centile) Time from onset to referral (< 24 h) – no. (%)	3.5 (14.3; 0–60) 23 (29.5)	0 (1.0; 0–10) 799 (60.0)	**< 0.001** **< 0.001**
Clinical presentation – no. (%) - Head deviation - to the right - to the left - Pain - Sore throat - Fever (> 38 °C) - Cervical tumefaction/lymphadenopathy - Headache - Earache - Vomiting - Dizziness/vertigo - Other	62 (79.5) 24 (38.7) 33 (53.2) 62 (79.5) 13 (16.7) 14 (17.9) 6 (7.7) 9 (11.5) 2 (2.6) 10 (12.8) 4 (5.1) 19 (24.4)	1117 (83.9) 461 (41.3) 401 (35.9) 1115 (83.7) 212 (15.9) 145 (10.9) 85 (6.3) 63 (4.7) 67 (5.0) 34 (2.6) 15 (1.1) 106 (8.0)	0.303 0.690 **0.006** 0.586 0.865 0.157 0.631 **0.015** 0.584 **< 0.001** **0.018** **< 0.001**
Laboratory workup - WBC (cells/µl) – median (IQR; 5° – 95°) - Neutrophils (%) – median (IQR; 5° – 95°) - Leukocytosis – no. (%)* - CRP ≥ 0.5 mg/dl – no. (%)*	9350 (7240; 4010–21,500) 62.0 (21.1; 31.5–88.1) 10 (28.6) 16 (50.0)	9530 (5030; 5641–18,538) 57.0 (27.6; 21.4 − 83.4) 58 (31.4) 89 (49.7)	0.789 **0.008** 0.856 1.000
Medical imaging – no. (%) - Radiography with findings - CT scan with findings - Ultrasound with findings - MRI with findings	75 (96.2) 65 (83.3) 60 (76.9) 37 (47.4) 34 (43.6) 15 (19.2) 7 (9.0) 26 (33.8) 21 (26.9)	587 (44.1) 110 (8.3) 22 (1.7) 39 (2.9) 8 (0.6) 150 (11.3) 104 (7.8) 369 (27.8) 61 (4.6)	**< 0.001** **< 0.001** **< 0.001** **< 0.001** **< 0.001** **0.034** 0.712 0.256 **< 0.001**
Specialist consultation – no. (%) - Orthopedic - Neurologist - Otolaryngologist - Neurosurgeon - Ophthalmologist - Oncologist - Anesthesiologist - Other	69 (88.5) 44 (56.4) 18 (23.1) 9 (11.5) 26 (33.3) 11 (14.1) 15 (19.2) 3 (3.8) 11 (14.1)	290 (21.8) 188 (14.1) 94 (7.1) 40 (3.0) 11 (0.8) 24 (1.8) 1 (0.1) 4 (0.3) 18 (1.4)	**< 0.001** **< 0.001** **< 0.001** **< 0.001** **< 0.001** **< 0.001** **< 0.001** **0.005** **< 0.001**
Etiology – no. (%) - Postural - Traumatic - Infective - Neurologic - Oncologic - Congenital - Other	0 (0) 35 (44.9) 15 (19.2) 8 (10.3) 17(21.8) 0 (0) 3 (3.9)	607 (45.6) 380 (28.5) 254 (19.1) 38 (2.9) 0 (0) 35 (2.6) 17 (1.3)	**< 0.001** **0.002** 0.974 **0.003** **< 0.001** 0.257 0.118
Treatment of torticollis – no. (%) - Ibuprofen - Acetaminophen - Antibiotics - Cervical collar - Surgery – no. (%)	63 (80.8) 45 (57.7) 4 (5.1) 21 (26.9) 45 (57.7) 18 (23.1)	1192 (89.6) 1076 (80.8) 107 (8.0) 104 (7.8) 761 (57.2) 0 (0)	**0.016** **< 0.001** 0.354 **< 0.001** 0.928 **< 0.001**
- Hospitalization – no. (%) - Hospitalization (days) – median (IQR; 5° − 95°) - Sequelae – no. (%)	61 (78.2) 7 (11; 1–39) 18 (23.1)	95 (7.1) 3 (4; 1–12) 11 (0.8)	**< 0.001** **< 0.001** **< 0.001**

*The percentages were calculated accounting for missing values

Patients with an urgent condition underlying the torticollis were significantly older (median age 6.9 vs. 5.7 years, *p* = 0.027), more frequently reported a history of trauma (41% vs. 27.2%, *p* = 0.008), and were admitted to the ED significantly later than patients without an urgent condition (23% vs. 60% in less than 24 h, *p* < 0.001). Regarding clinical presentation, a left head deviation was more frequent in patients with an urgent condition than in those without (53.2% vs. 35.9%, *p* = 0.006). Similarly, headache (11.5% vs. 4.7%, *p* = 0.015), vomiting (12.8% vs. 2.6%, *p* < 0.001) and dizziness (5.1% vs. 1.1%, *p* = 0.018) were more prevalent in patients with urgencies. Considering the diagnostic work-up, as expected, instrumental investigations (96.2% vs. 44.1%, *p* < 0.001) and specialist consultations (88.5% vs. 21.8%, *p* < 0.001) were performed more frequently in patients with urgent causes of torticollis, with also more frequent pathological findings (see Table 3). Antibiotics (26.9% vs. 7.8%, *p* < 0.001) and ibuprofen (57.7% vs. 80.8%, *p* < 0.001) were administered more frequently in patients with urgent and non-urgent conditions, respectively. Hospitalization (78.2% vs. 7.1%, *p* < 0.001), with a longer median duration (7 vs. 3 days, *p* < 0.001) was observed more frequently in patients with urgent causes of torticollis. Sequelae were described more in patients with urgent conditions (23.1% vs. 0.8%, *p* < 0.001).

The logistic regression analysis, adopting urgency of the underlying condition as the dependent variable, is shown in Table 4.

**Table 4:** Logistic regression analysis (dependent variable: urgent cause of torticollis)

Variables	OR	CI 95%	p–value
History of trauma (yes)	1.575	0.968–2.563	0.068
Time from onset to referral (days)	0.985	0.975–0.996	**0.006**
Headache (yes)	2.447	1.162–5.152	**0.018**
Vomiting (yes)	3.633	1.580–8.353	**0.002**

We found that time from onset of torticollis to referral to the ED (OR 0.98, p 0.006) was negatively associated with the odds an underlying urgent condition in the logistic model. By contrast, the presence of clinical signs/symptoms such as headache (OR 2.45, p 0.018) and vomiting (OR 3.63, p 0.002)) were the only variables strongly characterized by the presence of an urgent condition, after adjusting for the other clinically and statistically significant variables in the bivariate analysis.

## Discussion

This study represents the largest case series reported in the literature to date describing torticollis as a reason for admission to the pediatric ED. Comparing the results of our study with the two most similar studies, conducted by Pharisa et al. [4] and Starc et al. [5], we found a lower median age and confirmed a slight male prevalence. The incidence of torticollis was lower (1/522 accesses) than in the study by Pharisa et al. [4] (1/177 accesses out of 30,000 patients/year for 1 year) and by Starc et al. [5] (1/306 accesses out of 20,000 patients/year for 6 years). Regarding presenting symptoms, we observed a distribution similar to that found in the study by Starc et al. [5], with the exception of a lower prevalence of laterocervical tumefactions/lymphadenopathy in our study. Additionally, our patients underwent regional CT-scans more frequently, likely reflecting the absence of standardized guidelines for the management of pediatric torticollis leading to heterogenous diagnostic approaches. The overall distribution of causes mirrored that of the case series conducted in Trieste^5^, revealing a distinct predominance of postural etiology. However, in our study, patients with a history of abnormal neck movements were categorized under traumatic etiology. The detailed comparison of our study’s results with the studies by Pharisa et al. [4] and Starc et al. [5] is reported in the Supplementary Material.

Upon comparing patients with and without an underlying urgent condition, we found that those with an “urgent torticollis” tended to be slightly older and had delayed admission compared to patients with non-urgent conditions. However, this trend was reversed when examined through the multivariate model and weighted for the contribute of other relevant variables. Despite the significance of the result (*p* = 0.006, probably owing to the very narrow confidence interval), only a slight reduction of the OR was observed. Conversely, a history of trauma was also associated with a positive OR for an urgent cause of torticollis, albeit without reaching statistical significance. This finding likely reflects the inclusion of various post-traumatic disorders, such as cervical fractures or atlanto-epistrophic dislocation, within the category of urgent conditions. A few symptoms, mainly headache and vomiting, were associated with an urgent condition. This correlation was confirmed also in the multivariate analysis, were headache and vomiting were found to confer a 2.4- and 3.6-increase of the odds of a an underlying urgent condition, respectively. Indeed, these symptoms should raise the concern for an underlying neurological disorder causing intracranial hypertension. Unsurprisingly, cases necessitating imaging and referral to a specialist predominantly involved urgent underlying conditions. Analogously, antibiotic therapy and surgery were less frequently administered to patients with non-urgent causes of torticollis, who were more commonly prescribed ibuprofen as treatment.

The multivariate logistic regression model revealed that only headache and vomiting exhibited significant correlation with urgency, while a longer duration from onset to referral reduced the odds of an urgent condition. This is quite different from what Starc et al. [5], where the logistic regression analysis proved that fever, pharyngodynia, headache, and age less than 5 years were associated with hospitalization. Moreover, in our bivariate analysis, older age was associated with urgency. This discrepancy was likely attributed to the utilization of hospitalization as an outcome, which may be influenced by some factors other than the actual severity of the disease, including the increased caution often applied to younger patients. Unlike previous studies, our aim was to identify characteristics that could predict serious and potentially life-threatening conditions in order to diversify the diagnostic-therapeutic approach according to them.

**Fig. 2:**
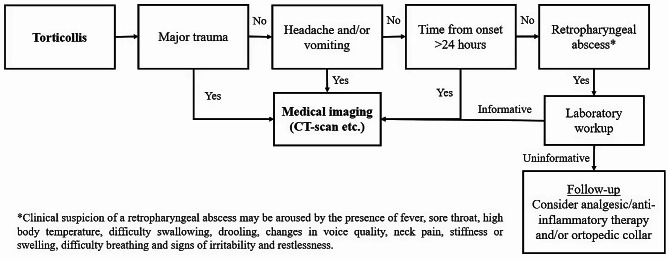
Proposed algorithm for torticollis

Considering the aforementioned variables, we propose the flow-chart (Fig. 2) outlining the management of patients with torticollis in the ED in the absence of other independent indications for admission and advanced imaging.

The retrospective design of the study represents a potential diagnostic bias, which is further increased by the vast heterogeneity of the clinical characteristics of our sample, collected over many years. Furthermore, our data, although conspicuous, originate from a single center, potentially constraining the reproducibility of our findings. Nonetheless, this is the largest report on pediatric torticollis in the current literature, with data partially conflicting with the existing ones, that requires further validation through future prospective studies.

## Conclusion

Our study represents the largest case series of patients admitted to the ED due to torticollis. It’s important to note that torticollis is a symptom, not a diagnosis. Further advanced imaging is warranted in patients with headache, vomiting and torticollis for more than 24 h, or who have not responded to analgesic/anti-inflammatory therapy or to application of an orthopedic collar. In any case, short-term clinical follow-up is recommended to re-evaluate the patient’s clinical condition and response to treatment, ensuring the symptom is effectively resolved.

### Electronic supplementary material

Below is the link to the electronic supplementary material.

Supplementary Material 1

## Data Availability

The datasets generated and analysed during the current study are not publicly available due privacy protection but are available from the corresponding author on reasonable request.

## Abbreviations

ED: Emergency Department
SCM: SternoCleidomastoid Muscle (SCM), OR = Odds Ratio
CI: Confidence Interval
