# Supplementary material for: Pediatric torticollis: clinical report and predictors of urgency of 1409 cases
Source: Ital J Pediatr. 2024 Apr 24;50:86. doi: 10.1186/s13052-024-01653-6 (PMC11044417; doi:10.1186/s13052-024-01653-6)
Supplement: Supplementary file 1 — Supplementary Material 1 [file 13052_2024_1653_MOESM1_ESM.doc]

Rome, March 3, 2024

**To:**

**Editor in Chief of The *“Italian Journal of Pediatrics”***

Prof. Giovanni Corsello,

University of Palermo, Palermo, Italy

**To:**

**Associate Editor of The *“Italian Journal of Pediatrics”***

Prof. Luigi Titomanlio,

Paris Cité University - Robert Debré Hospital APHP, Paris, France

Dear Prof. Corsello,

Dear Prof. Titomanlio,

here we are resubmitting an Original Research entitled “*Pediatric torticollis: clinical report and predictors of urgency of 1409 cases* by Umberto Raucci, Marco Roversi, Alessandro Ferretti, Valerio Faccia, Giacomo Garone, Fabio Panetta, Carlo Mariani, Eloisa Rizzotto, Antonio Torelli, Giovanna Stefania Colafati, Angelo Gabriele Aulisa, Pasquale Parisi, Alberto Villani, for the publication in *The “Italian Journal of Pediatrics”.*

We thank you and the reviewers for appreciation of our work.

The revision was carried out as requested by the reviewer: “The manuscript is very interesting .It deals wih a not so common condition ,The study is mainly an analysis of hospital data. The statistical analysis is meticulous and provides an insight to causes of Torticollis.
As it is a retrospective analysis of data ,the exact pathology cant be ascertained. The results are depicted with appropriate statistics.”. The following paragraph was modified at the end of the Discussion section and highlighted in red: “The retrospective design of the study represents a potential diagnostic bias, which is further increased by the vast heterogeneity of the clinical characteristics of our sample, collected over many years.”

We stated that the manuscript is being submitted only to *The “Italian Journal of Pediatrics”*, that it will not be submitted elsewhere, while under consideration, that it has not been published elsewhere, and, should it be published in The *“Italian Journal of Pediatrics*”, that it will not be published elsewhere—either in similar form or verbatim—without permission of the editors.

All authors are responsible for reported research.

The authors of the manuscript have no financial relationships to this article to disclose. Furthermore, the authors have no conflict of interest to disclose. No honorarium, grant, or other form of payment was given to anyone to produce the manuscript.

We hope that this article may be suitable for publication in *The “Italian Journal of Pediatrics”.* Many thanks for considering our article.

We look forward to hearing from you.

Yours sincerely,

Umberto Raucci for all other Authors.

**Corresponding address:**

[umberto.raucci@opbg.net](mailto:umberto.raucci@opbg.net)

Umberto Raucci, MD, PhD,

General Pediatrics and Emergency Department 2nd Level

Bambino Gesù Children’s Hospital, IRCCS,

Sant. Onofrio Place 4, Rome, 00165, Italy
